# Multi-Centre Clinical Evaluation of Photothermal Radiometry and Luminescence Correlated with International Benchmarks for Caries Detection

**DOI:** 10.2174/1874210601711010636

**Published:** 2017-12-19

**Authors:** Josh D. Silvertown, Stephen H. Abrams, Koneswaran S. Sivagurunathan, Julia Kennedy, Jinseok Jeon, Andreas Mandelis, Adam Hellen, Warren Hellen, Gary Elman, Richard Ehrlich, Raffy Chouljian, Yoav Finer, Bennett T. Amaechi

**Affiliations:** 1 Quantum Dental Technologies Inc, Toronto, Ontario, Canada; 2 Cliffcrest Dental Office, Scarborough, Ontario, Canada; 3 Center for Advanced Diffusion Wave and Photoacoustic Technologies (CADIPT), University of Toronto, Ontario, Canada; 4 Downsview Plaza Dental Office, Toronto Ontario, Canada; 5 Elm Tree Dental, Caledon, Ontario, Canada; 6 Scarborough North Dental Group, Toronto, Ontario, Canada; 7 Faculty of Dentistry, University of Toronto, Toronto, Ontario, Canada; 8 Institute of Biomaterials and Biomedical Engineering, University of Toronto, Toronto, Ontario, Canada; 9 University of Texas Health Science Center, San Antonio, Texas, USA

**Keywords:** PTR-LUM, ICDAS-II, The Canary System, Canary Number, Caries lesion, Diagnostic

## Abstract

**Introduction:**

A clinical study was initiated to investigate a caries detection device (The Canary System (CS)), based on photothermal radiometry and modulated luminescence (PTR-LUM). The primary objective of this study was to determine if PTR-LUM values (in the form of Canary Numbers; CN) correlate with International Caries Diagnostic and Assessment System (ICDAS II) scores and clinical situations. The secondary objectives of this study were to monitor the safety of PTR-LUM, and collect data to determine how CN values could be used to differentiate healthy from decayed tooth surfaces on a normalized scale.

**Methods:**

The trial was a four site, non-blinded study. Data was collected from 92 patients, resulting in 842 scanned tooth surfaces over multiple appointments. Surfaces were assessed according to ICDAS II, and further stratified into five clinical situation categories: 1) healthy surface, 2) non-cavitated white and/or brown spots; 3) caries lesions; 4) cavitation and 5) teeth undergoing remineralization therapy.

CN data was analyzed separately for smooth and occlusal surfaces. Using a semi-logarithmic graph to plot raw CN (rCN) and normalized (CN) values, rCN data was normalized into a scale of 0-100.

**Results:**

Linear correlations (R^2^) between CN and ICDAS II groupings for smooth and occlusal surfaces were calculated as 0.9759 and 0.9267, respectively. The mean CN values derived from smooth (20.2±0.6) and occlusal (19±1.0) surfaces identified as healthy had significantly lower CN values (*P*<0.05) compared with the values from the other clinical situation categories. No adverse events were reported.

**Conclusion:**

The present study demonstrated the safety of PTR-LUM for clinical application and its ability to distinguish sound from carious tooth surfaces. A clear shift from the baseline in both PTR and LUM in carious enamel was observed depending on the type and nature of the lesion, and correlated to ICDAS II classification codes, which enabled the preliminary development of a Canary Scale.

## 1 INTRODUCTION

The traditional methods for dental caries detection, visual, tactile and radiographic examinations, are not effective in detecting early caries lesions, particularly on occlusal surfaces [1-3]. Early detection of caries, before a substantial amount of the tooth is affected, can allow for minimally or non-invasive treatment; such as remineralization, mitigate radiation exposure, and result in a sound surface devoid of extensive restorations. Early caries detection technologies based on optical or electrical technologies include light-induced fluorescence, digitized imaging fiber-optic trans-illumination, alternating current impedance spectroscopy, and photothermal radiometry and modulated luminescence (PTR-LUM).

PTR-LUM is a non-invasive, energy conversion technology that monitors two different phenomena: 1) modulated thermal infrared radiation (PTR), and 2) alternating current luminescence (LUM) [4]. When laser light modulated at a fixed frequency is focused on a tooth, the tooth emits radiation (glows) at the same frequency, following radiative conversion of part of the incident optical energy to longer wavelength (Stokes shifted) LUM, and simultaneously releases heat at the same frequency, in the form of thermal infrared photons (“blackbody” or Planck radiation) following non-radiative conversion of the remaining incident energy. The analysis of the emitted radiation and of the emitted infrared photons, provides combined optical and thermal (“photothermal”) information about the condition of the crystal structure of the tooth. As a wave-based phenomenon, both PTR and LUM consist of amplitude and phase. In terms of the PTR signal, the amplitude (PTR-A) refers to the overall signal magnitude during the period the laser light shines on the tooth. The phase (PTR-P) represents the delay in the photothermal signal collected by the infrared detector with respect to a reference signal [5-10]. In addition to heat, a complementary signal channel, modulated LUM (in the forms of LUM-A and LUM-P), monitors the optical-to-radiative energy conversion, where the laser light is absorbed, by chromophore molecues which are raised to a higher-energy state, and longer wavelength luminescent light is emitted following their de-excitation. As a purely light based technique, its depth resolution in enamel is significantly limited by the high scattering coefficients of sound and carious enamel. Because thermal energy does not scatter like optical energy, PTR can reach deeper areas in the tooth than LUM alone [11]. As lesion severity increases (increased demineralization), there is a corresponding change in the amount of infrared radiation and luminescence collected. As remineralization progresses, a signal reversal trend indicates an improvement in the structural organization of the tooth. The key advantage of PTR-LUM is that caries lesions have optical absorption coefficients higher than healthy dental enamel, thereby ensuring high contrast against a minimally or non-absorbing healthy dental enamel. This simple fact can enhance the signal dynamic range increasing the sensitivity, thus making photonic devices using PTR-LUM suitable for detection of small lesions [4].

A commercially-available caries detection device (The Canary System^®^, CS) employs PTR-LUM technology using a low-power laser diode (<45 mW at the tooth surface) at 660 nm and modulated at 2 Hz [12]. Research has demonstrated that PTR-LUM has the potential to help oral health professionals detect and diagnose caries lesions and defects ≤ 5 mm below the enamel surface [11, 13, 14], occlusal pit and fissure caries [14, 15], smooth surface caries [9, 16], erosion lesions [17, 18], root caries [19], interproximal caries lesions [3, 20-22], caries around margins of restorations [23, 24], caries beneath the intact margins of amalgam restorations [25] and demineralization and remineralization of early caries lesions [5, 19, 26-29].

In 2008, a clinical study (QDT-101) was initiated to explore the safety and optimal scanning conditions for CS in 50 subjects [30]. In 2010, a follow-on clinical study (QDT-201, the focus of this manuscript) was initiated to evaluate how PTR-LUM performs *in vivo* in dental clinical practice situations and preliminary reports have been presented previously [31-33]. Specifically, the aim of the current study was to determine whether PTR-LUM values (in the form of Canary Numbers; CN) correlate with stages of tooth decay as defined by the International Caries Diagnostic and Assessment System (ICDAS II) and to develop the early foundation for a Canary Number scale by collecting data to determine how CN values could be used to differentiate healthy from decayed tooth surfaces on a normalized scale. The secondary objective of this study was to monitor the safety of PTR-LUM during application in dental practice.

## 2 MATERIALS AND METHODS

### 2.1 Study Design and Patient Selection

The trial was a four-site, non-randomized, non-blinded study. The investigators at each site were licensed dentists in Ontario, Canada (Supplemental Table **1**) who had been in clinical practice for at least 25 years. Each clinician received training in ICDAS ranking and two of the practices had prior experience in using ICDAS II to assess teeth [30]. The clinical trial monitor reviewed the ICDAS II criteria with each clinician periodically.

Patients of the site investigators’ clinics were invited to participate in this study. An assessment and screening visit was performed on 98 patients, which included questions on medical history, oral hygiene, and caries risk factors. Only subjects who met all inclusion criteria were eligible to participate in the study. Inclusion criteria were as follows: male or female subjects aged 18 years and older, inclusive, at the screening visit; that had been a registered patient at the dental clinic / investigational site for ≥ 6 months before the screening visit; a minimum of two teeth, with at least one healthy tooth surface; and sufficient knowledge of the English language. Subjects who met any exclusion criteria were excluded from participating in the study. Exclusion criteria were as follows: self-reported pregnancy, subjects using immunosuppressive drugs six months prior to the screening visit, and mental incapacity or language barriers which precluded consent, cooperation, or unwillingness to comply with the requirements of the trial protocol. After reviewing the inclusion and exclusion criteria, 92 patients were included in this 4 site clinical trial.

### 2.2 Ethics and Regulatory Review

This trial was conducted according to sections 83, 86, 87, and 88 of Health Canada’s Medical Device Regulations (MDR), 21 CFR Parts 50 & 56, 45 CFR Part 46, ICH Good Clinical Practice Guidelines, and the Canadian Tri-Council Policy Statement (TCPS). The study was approved under Health Canada’s MDR as an Investigational Testing Authorization (Class 2). Study protocol, related documentation and any amendments were reviewed and approved by an independent ethics review committee as protocol number QDT-201 (Institutional Review Board Services; Aurora, Ontario, Canada). Prior to performing any study-specific procedure, each subject was required to read and voluntarily sign a Research Ethics Board (REB)-approved informed consent form, indicating his/her free and informed consent to participate. This study was conducted as part of routine dental practice. Subjects’ routine dental care preceded the patients’ involvement in this trial and was based on the clinical needs of the subject as determined by the dentist using existing technologies in their respective practices. There were no treatments or interventions mandated as a result of this trial. At every visit, each clinical examiner screened the participant for adverse events, despite this study being the second clinical trial with the system [34]. A follow-up phone call / interview was done with each participant 24 hours after each visit. The procedures outlined for the assessment visit were repeated each time the subject came to the dental clinic as part of their routine dental care. There was no monetary compensation for participants in this trial.

### 2.3 Tooth and Tooth Surface Selection and Scanning

Site investigators were first trained on ICDAS II by a benchmark examiner and clinical trial monitor, using the training resources and tools found at the ICDAS Foundation website (www.icdas.org) and reviewed with the sites during the course of the trial. The clinical trial monitor reviewed the ICDAS II criteria with each site investigator periodically. Each investigator had chairside charts to help them with ICDAS II classification. Photographs were taken of each surface under examination. Notes were filed in clinical examination charts, indicating what was examined, ICDAS II number, PTR & LUM measurements for each tooth surface. This then allowed the team doing the data analysis to confirm that the ICDAS readings were correct.

Teeth and tooth surfaces selected for assessment in this study were selected for scanning at the discretion of the investigator at each site. The teeth underwent visual inspection and the surfaces were coded using the ICDAS II criteria [35], following plaque removal and 5 seconds drying of the surface using dental air-water syringe, particularly for the non-cavitated white/brown spot lesions. To be considered healthy for this study, a tooth surface must have scored an ICDAS II code of 0.

Tooth surfaces were classified into five clinical situation categories (Table **1**) by the investigator as follows: 1) healthy, 2) non-cavitated white spots and/or brown spots; 3) carious lesions; 4) cavitation, defined as caries that are to be restored/ treated on same day of scanning; and 5) teeth undergoing remineralization therapy. The monitoring data collected from the latter category (#5 – teeth undergoing remineralization therapy) will not be discussed in this manuscript but will be the basis for a subsequent report) but the CN and ICDAS II scores were included in the analysis.

The PTR-LUM device (The Canary System; Model: L-CS-CO-001; Quantum Dental Technologies Inc; Toronto, ON, Canada) was used to scan tooth sites according to the manufacturer’s instruction manual. Each patient’s scanning examination included a reference scan of a healthy surface (ICDAS code “0”) from the middle third of a maxillary central incisor since the area is the most commonly sound surface with at least 4 mm thickness of tooth tissue. The examiners were able to capture an image and assign a CN to that section of the tooth. All the CS data including raw data and images were stored on a cloud so that the data could be reviewed at a later date by the clinical trial monitors. The examination sites were dried with air before each CS scan.

### 2.4 Assessments and Device Outputs

At every visit, each clinical examiner screened the participants for adverse events. A follow-up phone interview was done with each participant 24 hours after each visit. Since the participants were registered patients of the examining dentist and attended clinic regularly safety was monitored at each subsequent visit to the dental practice even if it was not associated with the clinical trial. Device outputs used for evaluation included: PTR-amplitude (PTR-A) response; PTR-phase (PTR-P) response, LUM-amplitude (LUM-A) response, LUM-phase (LUM-P) response, raw (rCN) and normalized Canary Number (CN) values, and linear correlation with ICDAS II coding.

### 2.5 Calculation of Canary Number

Raw Canary Number (rCN) was calculated as follows: rCN = C x (PTR-A x PTR-P)/(LUM-A x LUM-P) (Equation **1**), derived from empirical observations of reliably consistent signal trends with changing health status of teeth [30]. To ensure that rCNs derived from tooth surfaces are consistent regardless of internal variability of detectors, optical and mechanical design, and electronic variability of each Canary device, rCNs were normalized [10, 36], where “C” is the local device calibration constant determined through a process known as “instrumental transfer function normalization” in the field of signal processing. “C” is derived by taking the ratio of the rCN measured from glassy carbon SIGRADUR G disks as calibration material (diameter 5 mm, thickness 5 mm), both sides lapped, one side diamond polished (R_a_<50 nm; HTW Hochtemperatur-Werkstoffe GmbH, Germany) between a reference Canary device and Canary devices located at each study site. The reason for using this material as a signal normalization reference was that its properties: optical (opaque; photothermally saturated, a condition meaning that its amplitude is maximum and is independent of the optical absorption coefficient), and geometric (essentially semi-infinite and thermally thick) were such that the theoretical PTR signal was independent of all those properties [6, 11, 15, 16] and thus any signal changes from device to device would be solely due to differences in instrumental responses, thereby determining transfer function of each individual device. Given that rCN can reach a theoretical maximum value of 1x10^4^, rCN values were converted into a normalized Canary Number (CN) using an arbitrary scale from 0-100. The range of rCN values that define a healthy/ sound surface was determined by setting the values derived from scanning tooth surfaces coded with an ICDAS II as “0”. As a preliminary method to determine the upper range that would indicate advanced decay, rCN values were derived from scanning glassy carbon, which is a black body absorber material the absorption coefficient of which is high enough at the Canary operating wavelength (660-nm), so that the PTR-A signal is photothermally saturated [10, 36], *i.e*. the material in optically opaque. Outliers statistically beyond two standard deviations were removed for this part of the analysis.

(1) $$\mathrm{CanaryNumber}\left(\mathrm{raw}\right)=\mathrm{rCN}=C\left(\frac{{\mathrm{PTR}}_{\mathrm{Amp}}•{\mathrm{PTR}}_{\mathrm{Phase}}}{{\mathrm{LUM}}_{\mathrm{Amp}}•{\mathrm{LUM}}_{\mathrm{Phase}}}\right)$$

### 2.6 Statistical Analysis

#### 2.6.1 Sample Size

The sample size calculations, which were based on a power analysis, were performed using nQuery Advisor software (Statistical Solutions). Based on the previous studies on PTR-LUM [3, 28], the number of patients recruited and subsequent surface scans acquired for this study was sufficient to yield a power of at least 95% for an effect size of 1.0. Calculations are based on standard unpaired t-tests, with two-tailed significance of α = 0.05.

#### 2.6.2 Demographic Data

Baseline demographic data for all subjects were summarized with descriptive statistics (mean, standard deviation, median, range and number of observations).

#### 2.6.3 Canary Scan Data

 The mean CN from five repeats for each scanned site on each tooth surface was calculated separately for smooth and occlusal surfaces for subsequent analysis. Normality tests followed by two-tailed t-tests with post-hoc analysis using a Bonferroni correction were performed to determine significance between clinical situations or ICDAS II. For easier analysis of parts of the data and given the limited number of available data for all 7 classes, ICDAS II scores were reclassified and grouped as follows: 0 (healthy), 1-2 (early caries), 3-4 (moderate caries), 5-6 (advanced caries). Precedents for collapsing ICDAS II scores into fewer categories have been reported [37, 38]. This grouping was used since the study was examining how clinicians would use PTR-LUM to develop a treatment approach for a particular lesion, and in line with the International Caries Classification and Management System (ICCMS^™^) [39]. Scan data derived from teeth with sealants, crowns, veneers, or teeth that were fractured were omitted from this part of the analysis.

## 3 RESULTS

### 3.1 Patient and Site Characteristics

Ninety-eight (98) patients were recruited and screened, and 92 met the inclusion criteria and were subsequently enrolled in the study. There were no patient drop-outs during the trial. Patients were recruited, screened and assessed from March 2010 to August 2011. Demographic data on these patients are outlined in Table **2**. Safety analysis concluded that there were no safety-related concerns related to CS; no AEs or ADEs were reported during or after the study. To determine if there were study site or patient age effects on the CN data, site-specific normalization constants were applied to the data: For example, study Site #4 had higher CN values on average, which could be attributed to the higher average age of patients at this site (mean = 55 years; range = 18-70 years) compared to the total study group age average of 45 yrs. Despite Study Site #1 having the majority of patients enrolled in this study (N=63), there were no other site-specific effects reported.

### 3.2 Normalizing Canary Numbers (CN) and Developing a Canary Scale

**Fig. (1):**
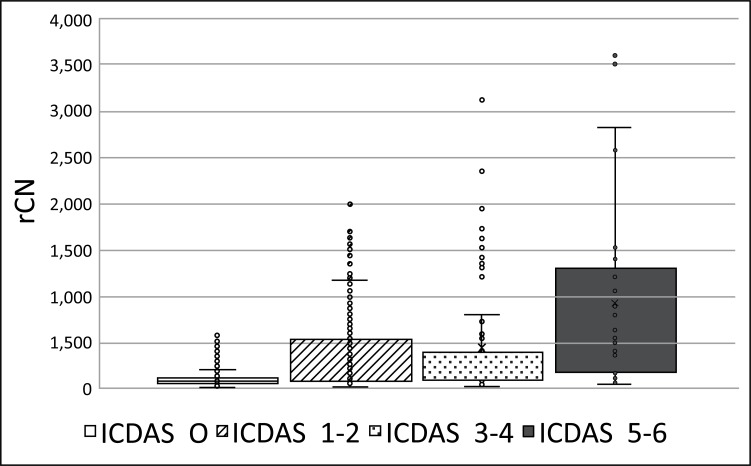
Box-Whisker plots showing distribution of all raw Canary Numbers (rCNs) by ICDAS II Grouping: 0 (healthy), 1-2 (early caries), 3-4 (moderate caries), 5-6 (advanced caries).

**Fig. (2):**
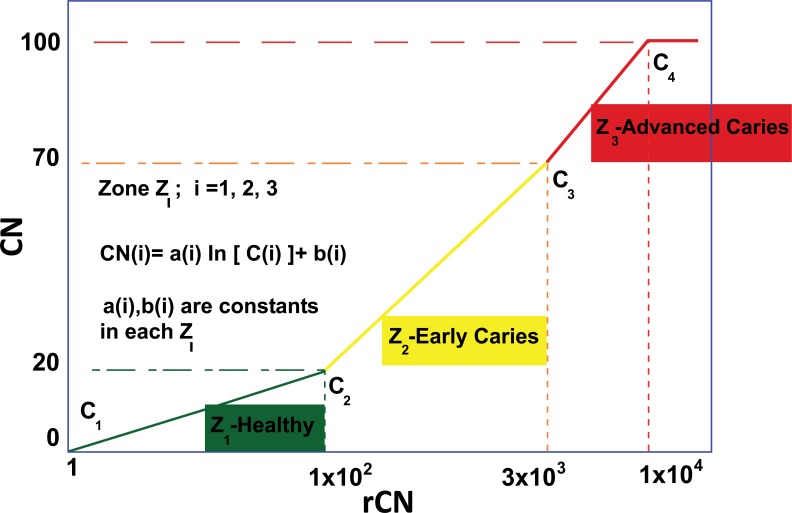
Semi-logarithmic plot used for the conversion of raw Canary Numbers (rCN) to normalized CNs for a universal 0 - 100 scale. CNs are determined from measured rCNs using the equation, *CN(i) = a(i)ln[rCN(i)] + b(i),* where *CN(i) =* Normalized Canary Number (an output value); r*CN(i)* = Raw Canary Number measured by The Canary System (an input value); i = 1 (Z_1_, healthy, CN = 0-20); i = 2 (Z_2_, suspicion of early caries, CN = 21-70); and i = 3 (Z_3_, suspicion of advanced caries, CN = 71-100). The coefficients *“a(i)”* and *“b(i)”* are the slope and y-intercept of the line in Zone (i), respectively. The variables *“a(i)”* and *“b(i)”* are unique to each zone.

Prior to Canary scanning, investigators performed a visual inspection of the tooth and applied a caries classification code according to ICDAS II. In order to determine the cut-off or threshold value for healthy surfaces, mean and standard deviations for rCN values by ICDAS II groupings were calculated (Supplemental Table **2**). The rCN mean and standard deviation values derived from 315 scanned surfaces (representing 83 patients) from surfaces classified as ICDAS II = 0 were 108.88 (±110.58). As the ICDAS ranking increased, so did the rCN mean values indicating that the higher the rCN the more advanced the caries. This distribution of rCNs for each ICDAS II Grouping is illustrated in a Box-Whisker plot showing this relationship (Fig. **1**). To determine the upper cut-off or threshold value for advanced decay surfaces, the mean rCN value from glassy carbon material was calculated to be 6,300 (Supplemental Fig. (**1**)). The relationship between rCN and CN values was plotted on a semi-logarithmic plot (Fig. **2**), where rCN data, shown on a logarithmic scale are linearly related to CN values with a universal scale of 0-100 to give oral health professionals a more precise, practical and clinically-relevant interpretive assessment. A graduated scale was implemented with three zones. Therefore, how rCN values are normalized into CN values depends on the slope of the lines between the three zones (Fig. **2**) calculated using Equation 2:

(2) $$\mathrm{Slope}\;\mathrm{of}\;\mathrm{the}\;\mathrm{line}\;\mathrm{in}\;\mathrm{Zone}\;\left(i\right)=a\left(i\right)=\frac{\left[{\mathrm{CN}\left(i\right)}_{\max}-{\mathrm{CN}\left(i\right)}_{\min}\right]}{\left[\ln\left(\frac{{\mathrm{rCN}\left(i\right)}_{\max}}{{\mathrm{rCN}\left(i\right)}_{\min}}\right)\right]}$$

Where, i = Zone 1, 2, 3,

i = 1 = Healthy Zone (0 – 20)

i = 2 = Early Caries Zone (21 – 70)

i = 3 = Advanced Caries Zone (71 – 100)

When plotted, a rCN of 1x10^2^ (rCN(1)_max_) representing the healthy cut-off of Z_1_ derived from scans for ICDAS II score 0 (rCN=108), a rCN of 3x10^3^ representing the minimum threshold of Z_3_ for advanced decay simulated from glassy carbon (rCN(2)_max_), and a rCN of 1x10^4^ representing the maximum rCN value (rCN(3)_max_), each represents the y-intercept boundaries [b (i)] for each of the three zones (Z_i_), calculated by Equation **3**:

(3) $$y-\mathrm{intercept}\;\mathrm{of}\;\mathrm{the}\;\mathrm{line}\;\mathrm{in}\;\mathrm{Zone}\;\left(i\right)=b\left(i\right)=\mathrm{CN}{\left(i\right)}_{\min}-a\left(i\right)\ln\left[\mathrm{rCN}{\left(i\right)}_{\min}\right]$$

The coefficients *‘a(i)’* and ‘*b(i)’* are unique to each zone and once determined through use of the normalizing material (glassy carbon) can be used to convert all rCN values into a CN.

(4) $$\mathrm{Normalized}\;\mathrm{Canary}\;\mathrm{Number}\;=\;\mathrm{CN}\left(i\right)=a\left(i\right)\ln\left(\mathrm{rCN}\left(i\right)\right)+b\left(i\right)$$

Values for Equations **2-4** for Zones i = 1, 2, 3 are summarized in Supplemental Table **3**.

To summarize, CN values can be calculated from rCN values using a three-step method Supplemental Fig. (**2**). As an example, a rCN value of 387.63 ± 413.45 (from Supplemental Table **2**) converts into a CN of 40 ± 11 (rounded to nearest integer; Supplemental Fig. (**2**)). Supplemental Table **2** shows rCN values converted into CN output values for each ICDAS II grouping. As expected, CN increases with ICDAS II code, indicating that CN correlates with severity of tooth decay. For the balance of the data analysis of this study, all rCN values were converted into CN values using the above methodology.

### 3.3 Correlating Canary Numbers by ICDAS II

**Fig. (3):**
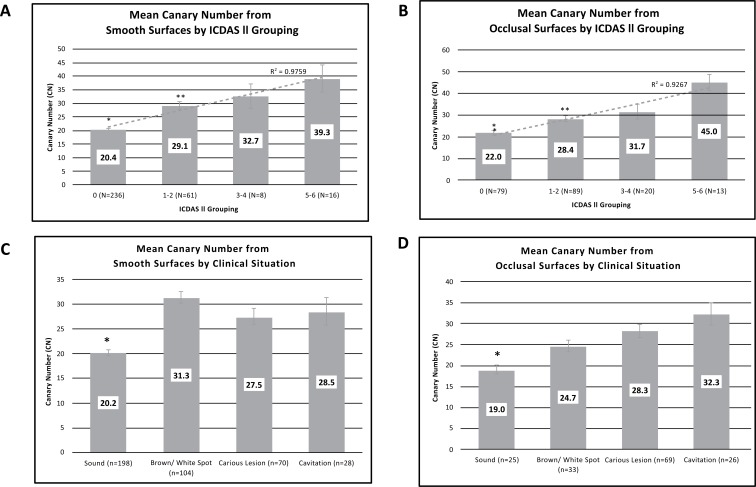
Mean Canary Numbers (CNs) for ICDAS II Groupings and clinical situation. Panels A and B). Mean Canary Numbers from all smooth (A) and occlusal (B) surface scans by ICDAS II Grouping: 0 (healthy), 1-2 (early caries), 3-4 (moderate caries), 5-6 (advanced caries). Single asterisk (*) indicates statistical significance (*P*<0.0083) between 0 (healthy) and all other groups. Double asterisk indicates statistical significance (*P*<0.0083) between the 1-2 (early caries) and the 5-6 (advanced caries) groups. Panels C and D). Mean Canary Numbers from all smooth (C) and occlusal (D) surface scans by clinical situation. Asterisk (*) indicates statistical significance (*P*<0.004) between sound and each of the other groups. “N” indicates the total number of scanned surfaces used in the calculation of each mean. Error bars represent the SEM.

Investigators classified tooth surfaces into clinical situation categories (Table **1**). Images were recorded for each tooth examined and the CN assigned to a section of the image that was being examined. Each clinical situation had a different number of patients who received scans in that category, where each patient had multiple smooth and/or occlusal surface scans (Table **1**). The mean CN value ranges for reclassified ICDAS II groupings (healthy surface, early, moderate, and advanced caries) are shown in Figs. (**3A** (for smooth surface) and **3B**) (for occlusal surface). The average CN values for smooth healthy surfaces (20.4±0.5) were significantly lower than those from the early caries (29.1±1.4; *P*<1x10^-12^), moderate caries (32.7±4.6; *P*<1x10^-5^), and advanced caries (39.3±5.1; *P*<5x10^-15^) groupings. Similarly, the average CN value ranges for healthy occlusal surfaces (22±1.1) were significantly lower than those from the early caries (28.4±1.3; *P*<1x10^-3^), moderate caries (31.4±3.4; *P*<1x10^-3^), and advanced caries (45.3±3.7; *P*<2x10^-11^) groupings. Linear correlations (R^2^) between CN and ICDAS II groupings for smooth and occlusal surfaces were calculated as 0.9759 and 0.9267, respectively and good agreement between the clinical judgement and CN for occlusal assessment (Fig. **3**).

### 3.4 Assessing Canary Numbers by Clinical Situation

Mean CN values collected for each clinical situation from all four study sites were calculated and amalgamated (Fig. **3C** and **3D**). As expected, CN values derived from smooth surfaces identified as healthy were significantly lower (20.2 ± 0.6) compared with mean CN values from surfaces identified as non-cavitated brown/white spot (31.3±1.3; *P* <2x10^-17^), caries lesion (27.5±1.6; *P*<3x10^-7^), or cavitation (28.5 ± 2.8; *P*<2x10^-5^). Similarly, CN values derived from occlusal surfaces identified as healthy were significantly lower (19 ± 1.0) compared with mean CN values from surfaces identified as non-cavitated brown/white spot (24.7±1.4; *P*<0.004), caries lesion (28.3±1.6; *P*<0.001), or cavitated (32.3 ± 2.6; *P*<3x10^-4^).

## 4 DISCUSSION

This study confirmed the safety and clinical effectiveness of a PTR-LUM based caries detection device as an adjunct tool for differentiating healthy from carious tooth surfaces. The reference standard in this study was the visual examination method using ICDAS II. A strong correlation was found between increasing CNs and ICDAS II classification, and statistially significant difference was found between the ICDAS II score 0 and the other severity score groups (1-2, 3-4, 5-6), as well as between the 1-2 *vs* 5-6 groupings. However, statistical significance was not found between the 1-2 *vs* 3-4 and 3-4 *vs* 5-6 groupings. There are several possible reasons to explain this. First, ICDAS II score 3 ranges from micro-cavitated early lesion to localized enamel breakdown, so teeth in those categories can yield similar readings. Although ICDAS II score 4 (underlying shadow from carious dentin) is not cavitated, it represents a wide range of dentin destruction underneath the enamel surface, which can be as severe as scores 5-6. Therefore, misclassification of sound surfaces as non-cavitated lesions, which has been reported in other studies [40], could explain the lower CN values than what would be expected. Given there were four clinical sites, each with its own site investigator, there could have been differences in the interpretation and/or coding using ICDAS II, especially with regards to ICDAS II score 3. Despite a standardized training on ICDAS II using provided resources and tools there were differences in the prior knowledge and experience with ICDAS II among the investigators. These differences could have resulted in investigators misclassifying samples. Two of the investigators had been involved in other studies using ICDAS II for lesion classification. It has been reported before that standardized training on ICDAS II can still result in lack of reliability in ICDAS coding [40]. This training discrepancy could also have been amplified due to the bias in data collected from Site #1 given that 68% of the patients were enrolled in that site. Second, the sample size for the 3-4 and 5-6 rankings were smaller than the ICDAS II code 0 and 1-2 groupings and this could result in higher variability among the samples. Lack of statistical significance between ICDAS II codes and groupings has been reported before when correlating caries detection aids with ICDAS II [41]. Third, while ICDAS II can be an effective and valid method for correctly diagnosing caries, other investigators have found that ICDAS II does not always correlate with lesion depth or histology [42-45]. One of the chief principles behind PTR-LUM technology is its ability to detect subsurface caries that are not visible. Given that ICDAS II coding is based on visual examination, it is possible that CS could have detected lesions that were not detected by the investigators when coding with ICDAS II, resulting in false positives [31].

Given the limitations of ICDAS II, tooth surfaces scanned with The Canary System in this study were classified into clinical treatment situations as another means to discern a trend in CN values: 1) healthy, 2) non-cavitated white spots and/or brown spots; 3) carious lesions; and 4) cavitation. Clinicians, using other caries detection technologies in their practices ranked the lesions according to how they would treat them. Similar to the trends observed with ICDAS II, CN values leading to ranking as “sound” were significantly lower than those in clinical situations that had visually-diagnosed pathology. This supports the utility of and basis for, using PTR-LUM as an objective technology to detect the presence / absence of caries.

## CONCLUSION

A key objective of this study was to obtain enough clinical data to enable the preliminary development of a Canary Scale comprised of CN values from 0-100. With 92 scanned patients resulting in a total of 842 scanned surfaces, a sufficient number of healthy tooth (ICDAS II score 0) surfaces (n=315) were scanned. After conversion of raw CN to normalized CN values, it was determined that a CN cut-off of ~20 could be used as the threshold to distinguish sound teeth from teeth with early caries. With an upper limit of a 70 (derived from glassy carbon as a uniform blackbody), a graduated Canary Number scale of 0-100 based on an empirical semi-logarithmic plot was developed. It was also found that in situations where both PTR-LUM and ICDAS II can yield data from caries lesions, there was a strong correlation between the two modalities. Data from the first clinical trial [32, 46] and in-vitro extracted tooth studies [31, 47] were used to help validate this Canary Number scale.

In summary, the results indicated that the PTR-LUM caries detection device used in this study is safe and is able to discriminate between healthy and carious enamel on smooth and occlusal surfaces. A clear shift from the baseline in both PTR and LUM in carious enamel was observed depending on the type, depth and nature of the lesion, according to ICDAS II classification codes, which enabled the preliminary development of a Canary Scale.

## Figures and Tables

**Table 1:** Clinical situation categories summarizing number of patient scans and surface scans.

**Clinical Situation**	**Number of Patients Scanned**	**Number of Smooth Surface Scanned**	**Number of Occlusal Surface Scanned**
**Healthy**	77	198	25
**White spots and / or brown spots**	49	104	33
**Caries**	60	70	69
**Cavitation – caries being treated same day as scanning**	23	28	26
**Teeth Undergoing Remineralization **	35	251	38

**Table 2:** Patient demographics for clinical trial.

**Patient Demographics **	
Total	92
**Age**	
Mean (STDEV)	45.3 (17.8)
Median	45.5
Min/Max	18/89
**Sex**	
Female	41 (44.6%)
Male	51 (55.4%)
**Ethic Origin**	
Black	4 (4.3%)
East Asian	3 (3.3%)
Hispanic/Latino	1 (1.1%)
South Asian	6 (6.5%)
White	78 (84.8%)
