# Supplementary material for: Multi-Centre Clinical Evaluation of Photothermal Radiometry and Luminescence Correlated with International Benchmarks for Caries Detection
Source: Open Dent J. 2017 Dec 19;11:636–47. doi: 10.2174/1874210601711010636 (PMC5738748; doi:10.2174/1874210601711010636)
Supplement: Supplementary file 1 [file TODENTJ-11-636_SD1.pdf]

Send Orders for Reprints to reprints@benthamscience.ae

BENTHAM OPEN

CrossMark

The Open Dentistry Journal

Supplementary Material

Content list available at: www.benthamopen.com/TODENTJ/

DOI: 10.2174/1874210601711010636

Multi-Centre Clinical Evaluation of Photothermal Radiometry and Luminescence Correlated with International Benchmarks for Caries Detection

Josh D. Silvertown[1], Stephen H. Abrams[1,2,*], Koneswaran S. Sivagurunathan[1], Julia Kennedy[1], Jinseok Jeon[1], Andreas Mandelis[1,3], Adam Hellen[1,2], Warren Hellen[2], Gary Elman[4], Richard Ehrlich[5], Raffy Chouljian[6], Yoav Finer[7,8] and Bennett T. Amaechi[9]

[1]*Quantum Dental Technologies Inc, Toronto, Ontario, Canada*
[2]*Cliffcrest Dental Office, Scarborough, Ontario, Canada*
[3]*Center for Advanced Diffusion Wave and Photoacoustic Technologies (CADIPT), University of Toronto, Ontario, Canada*
[4]*Downsview Plaza Dental Office, Toronto Ontario, Canada*
[5]*Elm Tree Dental, Caledon, Ontario, Canada*
[6]*Scarborough North Dental Group, Toronto, Ontario, Canada*
[7]*Faculty of Dentistry, University of Toronto, Toronto, Ontario, Canada*
[8]*Institute of Biomaterials and Biomedical Engineering, University of Toronto, Toronto, Ontario, Canada*
[9]*University of Texas Health Science Center, San Antonio, Texas, USA*

SUPPLEMENTARY TABLES AND FIGURES

Supplemental Table 1. Participating Study Sites.

Study Site #	Investigator Name	Office Address	Number of Patients Scanned
1	Dr. Stephen Abrams & Dr. Warren Hellen	Cliffcrest Dental Office 2995 Kingston Road, Scarborough, Ontario M1M 1P1, Canada	63
2	Dr. Gary Elman	Downsview Plaza Dental Office 1078 Wilson Avenue, Toronto, Ontario, M3K 1G6, Canada	8
3	Dr. Richard Ehrlich	Elm Tree Dental 17201 Highway 50 Palgrave, Ontario, L7E 3S9, Canada	5
4	Dr. Raffy Chouljian	Scarborough North Dental Group 404 Finch Avenue East Suite 204 Toronto, Ontario, M1S 4V5, Canada	16

Supplemental Table 2. Mean raw Canary Numbers (rCN) and normalized Canary Numbers (CNs) derived from patients scans.

ICDAS II Grouping	Raw Canary Number (rCN)		Normalized Canary Number (CN)		# of Scanned Surfaces
	Mean	SD	Mean	SD	
**0**	108.88	110.58	21	11	315
**1-2**	387.63	413.45	40	11	276
**3-4**	435.48	616.77	42	13	78
**5-6**	919.77	1006.34	53	10	29

Supplemental Table 3. Summary of parameters and corresponding values used for equations to convert raw Canary Numbers (rCN) into normalized Canary Numbers (CN).

Parameter	Value
$CN(1)_{min}$	0
$CN(1)_{max}$	20
$CN(2)_{min}$	20
$CN(2)_{max}$	70
$CN(3)_{min}$	70
$CN(3)_{max}$	100
$rCN(1)_{min}$	1
$rCN(1)_{max} = rCN(2)_{min}$	100
$rCN(2)_{max} = rCN(3)_{min}$	3,000
$rCN(3)_{max}$	10,000

$$CanaryNumber(raw) = rCN = C\left(\frac{PTR_{Amp} \bullet PTR_{Phase}}{LUM_{Amp} \bullet LUM_{Phase}}\right) \quad \text{(Equation 1)}$$

Where PTR_{Amp} = PTR Amplitude; PTR_{Phase} = PTR_{Phase}; LUM_{Amp} = Luminescence Amplitude; LUM_{Phase} = Luminescence Phase; and C: Instrumental Normalization Constant

PTR_{Amp} = 341.79 ± 4.3602 (uv)
PTR_{Phase} = 163.89 ± 0.77821 (degree)
LUM_{Amp} = 49.687 ± 1.1090 (uv)
LUM_{Phase} = 179 ± 0.037417 (degree)
C=1000

Using equation 1,
rCN = 6300

Supplemental Figure 1. Calculation of rCN for glassy carbon reference material.

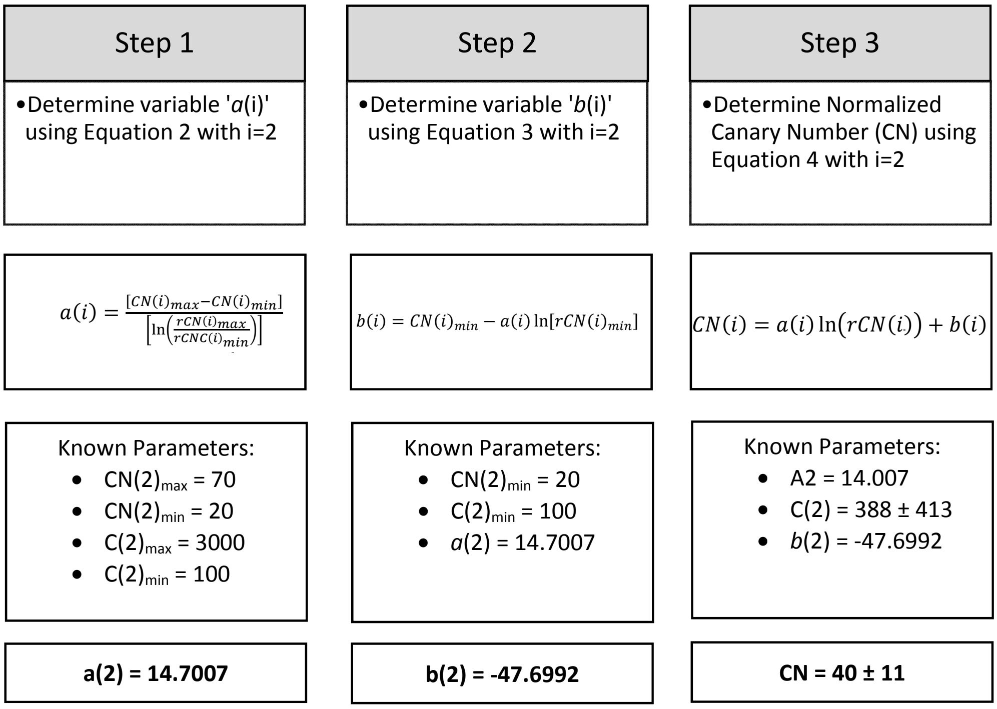

Supplemental Figure 2. Example of three-step process for converting a raw Canary Number (rCN) into a normalized Canary Number.

© 2017 Silvertown *et al.*

This is an open access article distributed under the terms of the Creative Commons Attribution 4.0 International Public License (CC-BY 4.0), a copy of which is available at: https://creativecommons.org/licenses/by/4.0/legalcode. This license permits unrestricted use, distribution, and reproduction in any medium, provided the original author and source are credited.
